# The Company of Biologists: a century in review

**DOI:** 10.1242/dmm.052792

**Published:** 2025-12-19

**Authors:** O. Claire Moulton, Saanjbati Adhikari, Rachel Hackett, Katie Ward

**Affiliations:** ^1^ Chief Executive Officer, The Company of Biologists; ^2^ Reviews Editor, The Company of Biologists; ^3^ Managing Editor, Disease Models & Mechanisms; ^4^ Chief Financial Officer and Charity Director, The Company of Biologists

## Abstract

**Summary:** In this Editorial, The Company of Biologists’ team look back over our celebrations of the Company's 100-year anniversary and directions for the future.

The year 2025 marks a significant milestone in the history of The Company of Biologists, the not-for-profit publisher of Disease Models & Mechanisms (DMM), and we have enjoyed an extraordinary year of celebration. We now take the opportunity to look back on activities that included our Biologists @ 100 conference in March and the content we've published throughout the year to celebrate our publishing and charitable activities. At the same time, we share a new strategic plan for The Company of Biologists, with a focus on supporting biologists and inspiring biology – for the next 100 years.

## A unique conference to celebrate community

A highlight of our anniversary year was the Biologists @ 100 conference, which took place in Liverpool from 24 to 27 March 2025. The meeting brought together researchers from across the disciplines represented by our five journals: Development, Journal of Cell Science, Journal of Experimental Biology, Disease Models & Mechanisms and Biology Open. It was held in partnership with the three societies we help support through our charitable funding – the British Society for Cell Biology (BSCB), the British Society for Developmental Biology (BSDB) and the Society for Experimental Biology (SEB). Altogether, we welcomed 585 attendees from 27 countries (and six continents), featuring research from 76 speakers and 286 posters.

The conference programme offered a unique opportunity to attend a diverse range of sessions. Topics were chosen to pique people's curiosity to learn something new in a talk they might not normally have chosen. In our plenary sessions – on climate change and biodiversity loss, health and disease, and emerging technologies – we wanted to tie together this curiosity-driven approach by setting our conference themes within a broader context, focusing on key challenges and opportunities facing biologists, and the world, today (https://www.biologists.com/100-years/conference/videos/). We thank our scientific organisers – Sarah Bray, DMM Editor Steve Clapcote, Craig Franklin, Steve Royle, Holly Shiels and Jim Smith – for their contributions to this interdisciplinary scientific programme.

The event itself was a showcase for sustainable conferencing, with carbon-conscious choices embedded in everything from venue selection and delegate travel through to plant-based catering and a sustainability zone. We took the opportunity to preview our Event carbon calculator (https://www.biologists.com/sustainability-hub/event-carbon-calculator/), which helps meeting organisers reduce the carbon footprint of their events. We were also delighted to be joined by the Woodland Trust, who support our biodiversity initiative, The Forest of Biologists (Moulton and Freeman, 2023).

We also took the opportunity to host author focus groups at the conference – asking the community to help us shape our journals for the future by sharing their publishing priorities, highlights and concerns. The Company is privileged to be run by a Board of Directors, who give their time to The Company of Biologists without payment. They are experienced, senior scientists from a range of life science and clinical research backgrounds. The Directors are keen to embrace change and experiment, and it is important that this is guided by the very real needs of the research community

## Confronting the challenge of antimicrobial resistance

As part of the Biologists @ 100 conference, DMM elected to host a day-long symposium focused on ‘Interdisciplinary approaches to combatting antimicrobial resistance’.

Antimicrobial resistance (AMR) poses a severe global health threat, driven by microorganisms such as viruses, bacteria and fungi evolving to resist the medicines designed to kill them, leading to drug-resistant strains. AMR is linked to rising morbidity, mortality and economic burden. Addressing this emergency requires a global ‘One Health’ approach, integrating human, animal and environmental health, alongside improved public awareness, surveillance and management, and, of particular relevance to DMM, research into new diagnostics, drugs and vaccines.

DMM's stated aim is to foster interdisciplinary collaboration and to serve as a platform for high-quality research that drives real-world impact. Given the clear need for action to combat AMR, DMM invited Editorial Advisory Board member Serge Mostowy and Katherine Duncan to organise a symposium that would bring together international experts to share insights from basic science to clinical applications. It was a fascinating and thought-provoking day; you can read more about it in an Editorial from organisers Serge and Katherine (Mostowy and Duncan, 2025), and a story from attendee Godwin Pius Ohemu.

## Celebrating our history and charitable impact

The centenary provided a perfect opportunity to review the 100-year history of our Company and the impact of our charitable funding, and to consider ways in which we can better support the community and the Company's mission in the future.

Over the past year, DMM has published articles as part of our anniversary subject collection. The year began with some of our Directors reviewing the journey of The Company of Biologists (Bray et al., 2025), and, in February, we looked back over the 18-year history of the journal (Adhikari and Hackett, 2025). It is remarkable how much has changed in such a short amount of time, from the perspective of disease models and publishing trends.

In addition, DMM published ‘A Model for Life’ interviews with Biologists @ 100 plenary speakers Sadaf Farooqi (Farooqi, 2025) and Charles Swanton (Swanton, 2025), two clinician–scientists at the forefront of tackling two major disease areas – obesity and cancer. Professor Sadaf Farooqi (University of Cambridge, UK) investigates the genetics underpinning obesity. Her research uncovered the first known genes that cause severe obesity, highlighting the significant role of appetite in regulating weight gain. Sadaf's work has been instrumental in proving that many observations in mice are also true in humans. As well as her research, Sadaf discussed her approach to exploring new research questions, and how these discoveries can ultimately impact patients and society. Professor Charles Swanton (The Francis Crick Institute, UK) works on understanding cancer evolution, with a particular focus on lung cancer. In his interview, we outline Charles’ fascinating discoveries at the interface of clinical and laboratory research, the challenges of translating science to the clinic and the future of cancer prevention.

As a not-for-profit organisation, The Company of Biologists has long supported scientists and learned societies, including, in particular, by providing financial support to early-career researchers through the Travelling Fellowships scheme and Conference Travel Grants (Hackett, 2025). Another key initiative, launched by the Company in 2010, is the Workshop programme, providing the organisational resources, funding and facilities to host small discussion meetings. In addition, in 2019, DMM joined its sister journals in organising important annual international meetings serving the communities around our journal. Aside from organising our own meetings, we offer Scientific Meeting Grants of up to £6000 to help cover the costs of organising in-person or virtual meetings, workshops and conferences in the fields represented by our journals.

As the Company moves into its next century, we remain deeply committed to advancing biological research, supporting the research community and fostering the next generation of scientists.

Making 2025 a year full of activity and celebration has relied on enthusiasm and effort from every member of our staff here at The Company of Biologists, including all our departments from Charity to Production, Sales & Marketing to Accounts, and Events to Editorial. We sincerely thank everyone for their contributions and you can learn more about some of our staff in our recent story ‘Celebrating the people behind the Company’. We also thank our colleagues who have collaborated across our five journals and three community sites to produce our anniversary content: Saanjbati Adhikari, Katherine Brown, Alejandra Clark, Amelia Glazier, Seema Grewal, Rachel Hackett, Michaela Handel, Kirsty Hooper, Kathryn Knight, Dina Mikimoto, Andrea Murillo and Helen Zenner. In addition, we thank the 100-anniversary project team, Jitske de Vries, Jane Elsom, Alex Eve, Claire Moulton and Katie Ward, who have overseen this anniversary year and all the ventures therein.

## Our extraordinary community

Throughout the year, we have spotlighted 100 biologists with an extraordinary link to the Company who have made an important contribution to biology (Box 1). ‘Extraordinary’ has many faces and it was difficult to restrict ourselves to 100 people, but this was a wonderful way to recognise a wide range of biologists from different career stages, locations and periods in history. Look out for our founder, the marine biologist George Parker Bidder III, in the collection, who at one point held the world record for the oldest message in a bottle (stemming from his work on ocean currents). To honour him, we set up our own ‘message in a bottle’ project. We've been gathering stories from you, our community, on how The Company of Biologists has supported you over time and how that has positively impacted your careers. Look out for more stories from the community on our dedicated web page.

Box 1. The ‘100 extraordinary biologists’ we have featured during 2025 Michael Abercrombie

Saanjbati Adhikari*

Aymen al-Rawi

Rohini Balakrishnan

Daniel Ríos Barrera

Renata Basto

Kênia Cardoso Bícego

George Parker Bidder III*

Anahí Binagui-Casas

Bob Boutilier

Andrea Brand

Sarah Bray

James Briscoe

Katherine Brown*

Carol Bucking

Clotilde Cadart

Paul Conduit

Kim Cooper

Mariana De Niz

Gautam Dey

Michael Dickinson

Jeroen Dobbelaere

Katherine Duncan*

Sadaf Farooqi*

Tony Farrell

Honor Fell

Jane Francis

Craig Franklin

Andrea Fuller

Joachim Goedhart

Daniel Gorelick

John Gurdon

Amanda Haage

Cathy Jackson

Helena Jambor

Monica Justice*

Steve Kelly

August Krogh

Tamina Lebek

Christophe Leterrier

Ottoline Leyser

James Liao

Grace Lim

Jennifer Lippincott-Schwartz

Sally Lowell

Emily Lucas

Simon Maddrell

Tshepiso Majelantle

Elaine Mardis*

Paul Martin*

Peter Medawar

Pleasantine Mill*

Mole

Claire Moulton*

Andrea Murillo*

Janni Nüsslein-Volhard

Godwin Pius Ohemu*

Guangshuo Ou

Hoover Pantoja-Sanchez

Sheila Patek

Andrea Paterlini

Liz Patton*

Mark Peifer

Norbert Perrimon*

Daisy Pineda-Suazo

Hans-Otto Pörtner

Manu Prakash

Reinier Prosée*

Jordan Raff

Liz Robertson

Nadia Rosenthal*

Janet Rossant*

Owen Sansom*

Martin Schwartz

Holly Shiels

Vivian Siegel*

Zolelwa Sifumba*

Jim Smith

Austin Smith*

Arnoud Sonnenberg

John Speakman

David Stephens

Kate Storey

Charles Swanton*

Stephen Tait*

John Treherne

Sylvie Urbé

Teresa Valencak

K. Vijayraghavan

Tobias Wang

Jennifer Waters

Fiona Watt

Michael Way

Vincent Wigglesworth

Caroline Williams

Chris Wood

Chris Wylie

Martha S. C. Xelhuantzi

Helen Zenner

Meng Zhu

Names with asterisks indicate biologists with specific links to DMM.

## Looking ahead – internal changes

**Fig. 1.**
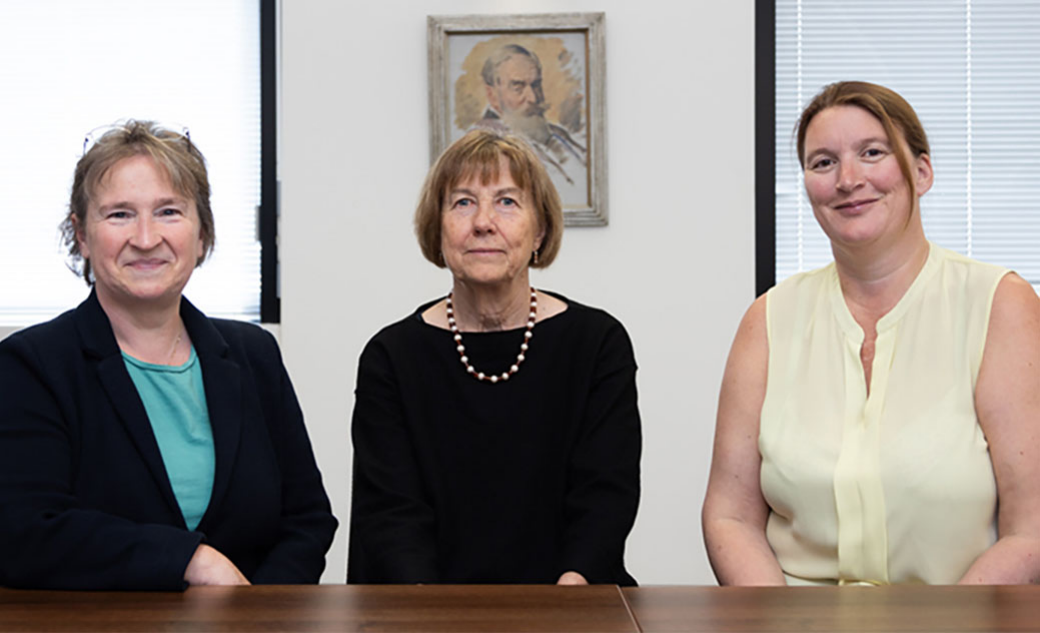
**A new structure for a new century.** From left to right: Claire Moulton (Chief Executive Officer), Sarah Bray (Chair of the Board of Directors) and Katie Ward (Chief Financial Officer and Charity Director).

The anniversary year also saw internal transformation as the Company turned its thinking towards the future. Claire Moulton was appointed as the Company's first Chief Executive Officer, with Katie Ward being appointed as Chief Financial Officer, reflecting a commitment from the Board of Directors to harness our in-house publishing expertise and promote collaborative thinking across the organisation (Fig. 1).

Our new strategic plan (2025–2028) focuses on four key areas. (1) Work together across all areas of the organisation to support the biological community, including building on our relationships with societies and other philanthropic organisations. (2) Strengthen our journals as highly respected publications that support the advancement of biological research while preserving the unique qualities of our journals, providing a great author experience and building trust and transparency in our publishing practices. (3) Develop a sustainable pathway to support the Company's activities for another 100 years, covering both financial sustainability and environmental sustainability. (4) Build on our reputation for community-focused innovation – 2025 featured Biology Open’s Fast & Fair peer review initiative and the sustainability team's Event carbon calculator.

As the anniversary year draws to a close, The Company of Biologists has had a lot to celebrate. The next 100 years will give us even more to think about as we continue our commitment to supporting biologists and inspiring biology.
